# Supplementary material for: Kinase KEY1 controls pyrenoid condensate size throughout the cell cycle by disrupting phase separation interactions
Source: bioRxiv. 2025 Oct 10:2025.10.09.681382. Preprint. [Version 1] doi: 10.1101/2025.10.09.681382 (PMC12632510; doi:10.1101/2025.10.09.681382)
Supplement: Supplement 2 [file NIHPP2025.10.09.681382v1-supplement-2.pdf]

bioRxiv preprint doi: https://doi.org/10.1101/2025.10.09.681382; this version posted October 10, 2025. The copyright holder for this preprint (which was not certified by peer review) is the author/funder, who has granted bioRxiv a license to display the preprint in perpetuity. It is made available under aCC-BY 4.0 International license.

# Supplementary Tables

**Supplementary Table 1. Immunoprecipitation-mass spectrometry (IP-MS) dataset, related to Fig 1.** The full dataset was used to generate Fig. 1b, c. EPYC1-Venus-3×FLAG or KEY1-Venus-3×FLAG were used as baits, and Venus-3×FLAG was used as a control bait to test for non-specific interactions. Attached as Excel.

**Supplementary Table 2. Oligonucleotides used in this work, Related to Methods.**

| Primer name | Sequence |
|---|---|
| CIB_3'_F | GACGTTACAGCACACCCTTG |
| CIB_5'_R | GCACCAATCATGTCAAGCCT |
| 3673_F | GTGGTACTTGATGGTCAGCC |
| 3676_F | GTACTTGATGGTCAGCCGG |
| 3785_R | CGCAGAACCCGAAGTCAG |
| 3809_R | CCCAAGCCCCCAAAATACC |
| 949_CLiP_F | TGCTACGACCCCAGCTACTT |
| 949_CLiP_R | CCAGATCCACCACTTCACCT |
| 4859-5018_F | ACCGAGCCTCCACCCCTAAC |
| 4859-5018_R | TAGGGCAGCCCCAGCATCAC |
| KEY1_473-491_adapter_F | GCTACTCACAACAAGCCCAGTTATGCGGCAAGCCACTCTTG |
| KEY1_5967-5984_adapter_R | GAGCCACCCAGATCTCCGTTCTTGGGCGAGCGGTAGCG |
| KEY1_US-2141_BstBI_F | GAGCAGGCGGCCTGGCTGTTCTACACACTTCCACACCACCAACC |
| KEY1_584_AgeI_R | GCGAGCGGATTTGACCGG |
| Key1_P1Fw | ACCGGGCAGGTGCTGTTC |
| Key1_p1Rv | CGTCGCGCTCGTACACTCG |
| CBLP FW | CAAGATCTGGGACCTGGAGAGC |
| CBLP RV | CTGGGCATTTACAGGGAGTGG |

**Supplementary Table 3. Plasmids used for transformation into Chlamydomonas and their resources, see also Methods.**

| Plasmid name | Gene | Promoter | Tags | Antibiotic Resistance in *E.coli* | Antibiotic Resistance in *Chlamydomonas* | Source |
|---|---|---|---|---|---|---|
| pLM005-EPYC1 | *EPYC1* (genomic DNA) | *psad* | Venus-3xFLAG | Ampicillin | Paromomycin | Mackinder et al., 2016 |
| pRAM118-pro+KEY1 | *KEY1* (genomic DNA) | *KEY1* (2kb upstream of ATG) | Venus-3xFLAG | Ampicillin | Hygromycin | This paper; submitted to Chlamydomonas Resource Center |
| pRAM118-pro+KEY1-SNAP | *KEY1* (genomic DNA) | *KEY1* (2kb upstream of ATG) | SNAP-3xFLAG | Ampicillin | Hygromycin | This paper; submitted to Chlamydomonas Resource Center |

bioRxiv preprint doi: https://doi.org/10.1101/2025.10.09.681382; this version posted October 10, 2025. The copyright holder for this preprint (which was not certified by peer review) is the author/funder, who has granted bioRxiv a license to display the preprint in perpetuity. It is made available under aCC-BY 4.0 International license.

| | | | | | | |
|---|---|---|---|---|---|---|
| pRAM118-pro+KEY1_ΔRBM-SNAP | *KEY1* (genomic DNA with mutated Rubisco-binding motif encoded sequence) | *KEY1* (2kb upstream of ATG) | SNAP-3xFLAG | Ampicillin | Hygromycin | This paper; submitted to Chlamydomonas Resource Center |
| pRAM118-proEPYC1-EPYC1-Venus | *EPYC1* (genomic DNA) | *EPYC1* (2kb upstream of ATG) | Venus-3xFLAG | Ampicillin | Hygromycin | This paper; submitted to Chlamydomonas Resource Center |
| pRAM118-proEPYC1-EPYC1-Astring-Venus | *EPYC1* (genomic DNA with all the Serine or Threonine encoding sequences changed to Alanine encoded sequences ) | *EPYC1* (2kb upstream of ATG) | Venus-3xFLAG | Ampicillin | Hygromycin | This paper; submitted to Chlamydomonas Resource Center |

**Supplementary Table 4. Chlamydomonas strains used in this study and their resources, see also Methods.**

| Chlamydomonas Resource Center ID | Strain description | Antibiotic Resistance | Plasmid used | Source |
|---|---|---|---|---|
| CC-4533 | cMJ030 (wild type) | none | none | Wildtype and parent strain to CLiP library |
| CC-1690 | Wild type mating type + | none | none | Chlamydomonas Resource Center |
| LMJ.RY0402.107748 | *key1-1* | Paromomycin | pMJ013b | CLiP mutant library |
| LMJ.RY0402.168949 | *key1-2* | Paromomycin | pMJ013b | CLiP mutant library |
| CC-6226 | *key1-1;RBCS1-Venus* | Paromomycin | pLM005-RBCS1 | This paper; by mating the strains key1-1 (mt-) and RBCS1-Venus (mt+), which is generated by mating the strain RBCS1-Venus (mt-) in a cMJ030 background with the wild-type strain CC-1690 (mt+). |
| CC-6227 | *key1-1;EPYC1-Venus* | Paromomycin, Hygromycin | pLM005-EPYC1 | This paper; by transformating the plasmid pLM005-EPYC1 into *key1-1* strain. |

bioRxiv preprint doi: https://doi.org/10.1101/2025.10.09.681382; this version posted October 10, 2025. The copyright holder for this preprint (which was not certified by peer review) is the author/funder, who has granted bioRxiv a license to display the preprint in perpetuity. It is made available under aCC-BY 4.0 International license.

| | | | | |
|---|---|---|---|---|
| CC-6228 | *key1-1;KEY1-Venus* | Paromomycin, Hygromycin | pRAM118-pro+KEY1 | This paper; by cloning and transforming the plasmid pRAM118-pro+KEY1 into *key1-1* strain. |
| CC-6229 | *key1-1;KEY1-SNAP* | Paromomycin, Hygromycin | pRAM118-pro+KEY1-SNAP | This paper; by cloning and transforming the plasmid pRAM118-pro+KEY1-SNAP into *key1-1* strain. |
| CC-6230 | *key1-1;RBCS1-Venus;KEY1-SNAP* | Paromomycin, Hygromycin | pRAM118-pro+KEY1-SNAP | This paper; by transforming the plasmid pRAM118-pro+KEY1-SNAP into *key1-1;RBCS1-Venus* strain. |
| CC-6231 | *key1-1;RBCS1-Venus;*$KEY1^{\Delta RBM}$*-SNAP* | Paromomycin, Hygromycin | pRAM118-pro+KEY1_ΔRBM-SNAP | This paper; by cloning and transforming the plasmid pRAM118-pro+KEY1_ΔRBM-SNAP into *key1-1;RBCS1-Venus* strain. |
| CC-6232 | *epyc1;EPYC1-Venus* | Paromomycin, Hygromycin | pRAM118-proEPYC1-EPYC1-Venus | This paper; by cloning and transforming the plasmid pRAM118-proEPYC1-EPYC1-Venus into *epyc1* strain. |
| CC-6233 | *epyc1;*$EPYC1^{phosphonull}$*-Venus* | Paromomycin, Hygromycin | pRAM118-proEPYC1-EPYC1-Astring-Venus | This paper; by cloning and transforming the plasmid pRAM118-proEPYC1-EPYC1-Astring-Venus into *epyc1* strain. |
